# Latent profiles of emotional intelligence and associated factors among clinical nurses: a cross-sectional study

**DOI:** 10.3389/fpubh.2026.1851059

**Published:** 2026-06-12

**Authors:** La Pan, Xiaofang Wu, Haowen Huang, Ting Liu, Ling Xia

**Affiliations:** 1 Department of Obstetrics and Gynecology, Affiliated Hospital of Jiangnan University, Wuxi, Jiangsu, China; 2 Wuxi School of Medicine, Jiangnan University, Wuxi, Jiangsu, China

**Keywords:** cross-section study, emotional intelligence, latent profile analysis, nurses, tertiary hospitals

## Abstract

**Background:**

Clinical nurses in tertiary hospitals face high emotional labor, which can deplete emotional resources and lead to occupational burnout. Emotional intelligence is a crucial psychological resource for coping with such stress. This cross-sectional study aimed to use latent profile analysis to identify emotional intelligence profiles among clinical nurses and examine factors associated with profile membership.

**Methods:**

A cross-sectional study was conducted between December 2025 and January 2026. A convenience sample of 765 clinical nurses from two tertiary Grade A hospitals in China was recruited. Data were collected using a demographic and work-related characteristics questionnaire and the Wong and Law Emotional Intelligence Scale. Latent profile analysis was used to identify distinct emotional intelligence subgroups, and multinomial logistic regression was performed to examine factors associated with profile membership.

**Results:**

Three distinct latent profiles of emotional intelligence were identified among the clinical nurses: “Dysregulated Low emotional intelligence” (41.7%), “Moderate-Balanced emotional intelligence” (50.8%), and “High-Balanced emotional intelligence” (7.5%). The majority of nurses exhibited an overall moderate level of emotional intelligence. Multinomial logistic regression revealed that parental overprotection or control, personality type, involvement in department management, and job satisfaction were significant factors associated with latent profile membership.

**Conclusions:**

The findings highlight the significant heterogeneity of emotional intelligence within the nursing workforce. Nursing managers may adopt differentiated management strategies and develop targeted training programs tailored to specific subgroup characteristics. Targeted support for emotional intelligence may contribute to nurses' mental wellbeing and the quality of nursing care.

## 1 Introduction

In China, tertiary Grade A hospitals represent the highest level within the national hospital classification system and provide comprehensive medical services for patients with complex conditions. Compared with lower-level healthcare institutions, these hospitals

are characterized by greater patient complexity, faster bed turnover, more specialized care demands, and higher requirements for interdisciplinary coordination (1, 2). Clinical nurses in tertiary Grade A hospitals therefore face substantial work demands, including technically complex nursing tasks, rapid responses to changing clinical conditions, and continuous attention to patients' emotional needs. Sustained heavy workloads and emotional labor may gradually deplete nurses' emotional resources and increase the risk of occupational burnout (3, 4). Studies have reported that the prevalence of burnout among nurses can reach 64.5% (5). Burnout can weaken nurses' capacity for empathy and humanistic care and increase the risk of nurse-patient communication difficulties and adverse nursing outcomes (6). In complex clinical situations, nurses must regulate their own emotions and respond to patients' emotional needs. Emotional intelligence (EI) has been recognized as an important psychological resource for coping with occupational stress and maintaining effective humanistic care (7). Higher EI levels are associated with better communication competence, higher job satisfaction, and improved patient safety outcomes (8), whereas lower EI levels are linked to burnout and insufficient humanistic care (9, 10).

Emotional intelligence has been broadly conceptualized as the capacity to perceive, understand, use, and regulate emotions in oneself and others (11). In the present study, EI was assessed using the Wong and Law Emotional Intelligence Scale (WLEIS), a self-report instrument that captures individuals' perceived emotional abilities across four dimensions: self-emotional appraisal, others' emotional appraisal, use of emotion, and regulation of emotion (12). These dimensions are closely relevant to nursing practice, where nurses must recognize patients' emotional cues, regulate their own emotional responses, and sustain therapeutic communication during care (13). A meta-analysis integrating structural equation modeling found that emotional labor was positively associated with job burnout, whereas EI was negatively associated with burnout (14). Evidence from structural equation modeling and intervention studies further suggests that EI may help nurses cope with work-related stress, reduce stress responses, and maintain communication competence in demanding clinical settings (15, 16). Empirical evidence further suggests that different dimensions of EI may not develop uniformly. Nurses who are frequently exposed to patients' suffering may become more sensitive to others' emotions and respond with empathy (17). However, when emotion use and regulation are relatively limited, heightened emotional perception may be accompanied by greater vulnerability to emotional exhaustion and compassion fatigue (18). Most previous studies have relied on total EI scores, which may obscure individual patterns across EI dimensions (19, 20).

Latent profile analysis (LPA) is a person-centered statistical approach that identifies distinct subgroups within a population based on individuals' response patterns across multiple variables, thereby revealing inherent heterogeneity among individuals (21). Several studies have applied LPA to examine EI among nurses in primary and secondary healthcare institutions (22, 23). However, because tertiary Grade A hospitals differ from lower-level healthcare institutions in patient complexity, care intensity, and emotional demands, EI profile structures and their associated factors identified in other nursing populations may not be directly generalizable to this workforce. Evidence regarding the latent profiles of EI among nurses working in tertiary Grade A hospitals remains limited. After identifying latent subgroups, examining the demographic and work-related characteristics associated with different profiles provides a basis for developing targeted interventions. Therefore, this study aimed to use LPA based on the four-dimensional WLEIS response patterns to identify latent EI profiles among tertiary Grade A hospital nurses and to explore demographic and work-related characteristics associated with latent profile membership. The findings may provide an empirical basis for nursing managers to design targeted EI enhancement strategies and promote both nurses' mental wellbeing and the quality of nursing care.

## 2 Materials and methods

### 2.1 Design

This study employed a cross-sectional design and followed the Strengthening the Reporting of Observational Studies in Epidemiology (STROBE) guidelines (24).

### 2.2 Setting and participants

A convenience sampling method was used to recruit clinical nurses from two tertiary Grade A hospitals in Wuxi City, Jiangsu Province, China, between December 2025 and January 2026. The inclusion criteria were: (a) holding a valid nursing license; (b) being currently employed during the survey period; and (c) voluntarily participating in this study. The exclusion criteria were: (a) being on sick or personal leave during the survey; and (b) being intern nurses or visiting nurses.

### 2.3 Sample size

The sample size was estimated separately for multinomial logistic regression and LPA. For the regression analysis, Kendall's rule of thumb recommends a sample size of 10–20 times the number of independent predictors to ensure stable parameter estimation (25). In this study, 19 demographic and work-related variables were considered as candidate predictors, requiring 190–380 participants. For LPA, previous methodological guidelines recommend a sample size of at least 500 participants to support stable model estimation (21). Ultimately, 765 valid questionnaires were collected, exceeding the recommended sample size for both multinomial logistic regression and LPA.

### 2.4 Instruments

The survey instruments included the demographic and work-related characteristics questionnaire and the WLEIS.

#### 2.4.1 Demographic and work-related characteristics questionnaire

Based on a literature review, the research team developed a questionnaire to assess demographic and work-related characteristics. The questionnaire consists of 19 items divided into two sections. The demographic characteristics section includes gender, age, marital status, birthplace, only child status, father's education level, mother's education level, history of parental overprotection or control, personality type (self-reported as extrovert, introvert, or ambivert), and educational level. The work-related characteristics section includes years of working experience, professional title, participation in specialty training, involvement in department management, gender of head nurse, years of head nurse experience, monthly income, income satisfaction, and job satisfaction. The measurement and coding of key self-developed predictor variables are presented in Supplementary Table S1.

#### 2.4.2 Wong and Law emotional intelligence scale (WLEIS)

The WLEIS was developed by Wong and Law (12) and validated for the Chinese context by Wang (26). It was employed in this study to assess nurses' self-reported EI across four dimensions. The scale consists of 16 items categorized into four dimensions: self-emotional appraisal (items 1–4), others' emotional appraisal (items 5–8), use of emotion (items 9–12), and regulation of emotion (items 13–16). Items are rated on a five-point Likert scale ranging from 1 (“strongly disagree”) to 5 (“strongly agree”). The total score ranges from 16 to 80, with higher scores indicating higher levels of EI. The scale has been used in Chinese nursing studies and has shown good reliability and validity (27). In this study, the Cronbach's alphas for the total scale, self-emotional appraisal, others' emotional appraisal, use of emotion, and regulation of emotion were 0.915, 0.831, 0.915, 0.839, and 0.906, respectively.

### 2.5 Data collection

Data collection was conducted between December 2025 and January 2026 using Questionnaire Star, a secure and professional online survey platform widely used in medical research in China. With the coordination of head nurses, the survey link and QR code were distributed to eligible nurses via internal WeChat groups. To ensure data quality, a pilot test was first conducted with 20 clinical nurses to verify the readability and clarity of the questionnaire items. During the formal survey, strict control measures were implemented: the platform was configured to allow only one submission per IP address, and all items were set as mandatory. Following data collection, two researchers independently screened the responses. Questionnaires were excluded if they exhibited consistent repetitive response patterns. Ultimately, 765 valid questionnaires were obtained from a total of 790, resulting in an effective response rate of 96.8%.

### 2.6 Data analysis

Data analyses were performed using Mplus 7.4 (Muthén & Muthén, Los Angeles, CA, USA), IBM SPSS Statistics 27.0 (IBM Corp., Armonk, NY, USA), and IBM SPSS Amos 28.0 (IBM Corp., Armonk, NY, USA). Prior to formal analysis, the data were inspected for accuracy. Since the online survey platform required all items to be answered, there were no missing data. Continuous variables were described using means and standard deviations (mean ± SD), and categorical variables were described using frequencies and percentages (*n*, %). Confirmatory factor analysis (CFA) was conducted using Amos 28.0 to verify the four-factor structure of the WLEIS within the current sample. Model fit was evaluated using the chi-square to degrees of freedom ratio (χ^2^/df), comparative fit index (CFI), Tucker-Lewis index (TLI), root mean square error of approximation (RMSEA), and standardized root mean square residual (SRMR). Acceptable model fit was indicated by χ^2^/df < 5.0, CFI and TLI > 0.90, alongside RMSEA and SRMR < 0.08. Standardized factor loadings were also examined to assess the association between each item and its corresponding latent factor. To assess the potential presence of common method bias, the unmeasured latent method construct (ULMC) approach was employed in AMOS 28.0. A latent common method factor was added to the measurement model, with loadings on all items and constrained to be uncorrelated with the substantive factors. Model fit was compared with that of the theoretical model to evaluate whether accounting for method variance improved model fit (28).

LPA was conducted using Mplus version 7.4 to identify distinct subgroups of nurses based on the four WLEIS dimensions. Models with one to five latent profiles were estimated using the robust maximum likelihood estimator. To reduce the risk of local maxima, 1,000 initial-stage random starts and 200 final-stage optimizations were used, with 20 initial-stage iterations. The bootstrap likelihood ratio test was conducted with 500 bootstrap draws. Model convergence and replication of the best log-likelihood value were examined to assess the numerical stability of the candidate solutions. Indicator variances were constrained to be equal across profiles, consistent with a parsimonious LPA specification. The four WLEIS dimension scores were entered into the LPA in their original scale metric because they were derived from the same instrument and measured using the same Likert-type response scale. This approach also facilitated interpretation of the mean patterns across profiles. Model selection was based on a combination of statistical fit, classification quality, class size, numerical stability, parsimony, and substantive interpretability. The following indicators were considered: (1) log-likelihood (LL), with larger values indicating better model fit; (2) information criteria, including the Akaike information criterion (AIC), Bayesian information criterion (BIC), and sample-size adjusted BIC (aBIC), with lower values indicating better model fit (21); (3) classification accuracy, assessed using entropy and average posterior probabilities (AvePP). Entropy ranges from 0 to 1, with values closer to 1 indicating greater classification certainty; values of 0.80 or higher suggest good classification quality (29). AvePP values of 0.70 or higher indicate adequate assignment of individuals to their most likely class (30); and (4) likelihood ratio tests, including the Lo-Mendell-Rubin adjusted likelihood ratio test (LMR) and the bootstrap likelihood ratio test (BLRT), which compare a model with *k* classes with a model with *k–*1 classes. A statistically significant *P*-value (< 0.05) indicates that the *k*-class model provides a better fit than the *k–*1-class model (21). In addition, the smallest class was required to include at least 5% of the total sample based on most likely profile membership (31).

Participants were classified into the latent profile with the highest posterior probability. Subsequent analyses were performed in SPSS 27.0. One-way analysis of variance (ANOVA) with Bonferroni correction was used to compare EI total and dimensional scores across profiles. Chi-square tests were used for unordered categorical variables. Kruskal–Wallis *H*-tests were used for ordinal categorical variables and continuous variables that were not normally distributed. Variables showing significant differences in the univariate analysis were considered for multinomial logistic regression. Before model construction, collinearity diagnostics were performed. Because age and years of working experience showed severe multicollinearity, with variance inflation factor values exceeding 20, they were not entered into the same model. Age was retained in the primary model because it had a relatively lower variance inflation factor and represents a fundamental baseline demographic covariate. Years of working experience was examined in a sensitivity analysis by replacing age.

To assess the potential influence of classification uncertainty in latent profile assignment, a sensitivity analysis was conducted. Participants were initially assigned to their most likely latent profile based on posterior probabilities. Individuals with a maximum posterior probability below 0.80 were then excluded to form a high-certainty subsample. The multinomial logistic regression was re-estimated in this subsample to assess the robustness of the core findings. All statistical tests were two-tailed, and a *P*-value of < 0.05 was considered statistically significant.

### 2.7 Ethical considerations

This study was approved by the Ethics Committee of The Affiliated Hospital of Jiangnan University (Approval No. LS2023077) and was conducted in accordance with the principles of the Declaration of Helsinki. Electronic informed consent was obtained from all participants prior to data collection. Before accessing the formal online questionnaire, participants were presented with a brief introduction regarding the aim and significance of the study to help them decide whether to participate. They were informed that their participation was voluntary and that they had the right to withdraw from the study at any time without any consequences. Participants indicated their consent by selecting the “Agree” option on the survey platform. Furthermore, all data were handled confidentially and anonymously to protect participants' privacy.

## 3 Results

Confirmatory factor analysis was conducted to examine the four-factor structure of the WLEIS in this sample. The model showed acceptable fit: χ^2^/df = 3.988, CFI = 0.963, TLI = 0.955, RMSEA = 0.063, and SRMR = 0.0437. The standardized factor loadings ranged from 0.50 to 0.93. These findings supported the use of the four WLEIS dimensions as indicators in the LPA.

The ULMC approach was utilized to screen for common method bias by comparing the theoretical measurement model with a model incorporating a latent common method factor. The changes in model fit were small (ΔRMSEA = −0.005, ΔSRMR = −0.0073, ΔCFI = 0.006, ΔTLI = 0.006). These differences indicated that adding the common method factor did not meaningfully improve model fit, providing limited evidence that common method bias substantially affected the measurement model.

### 3.1 Participant characteristics

A total of 765 nurses participated in this study. As shown in Table 1, most participants were female (95.0%), married (66.7%), and from rural areas (64.1%). The mean age was 33.45 ± 8.06 years. More than half of the participants were not only children (54.5%), and most held a bachelor's degree (84.4%). Fathers' and mothers' education levels were mainly junior high school or below. In addition, 21.6% of participants reported a history of parental overprotection or control. The most common personality type was ambivert (60.1%), followed by introvert (23.7%) and extrovert (16.2%). Regarding work-related characteristics, the mean years of working experience was 11.67 ± 8.81 years. Professional titles were mainly junior (49.9%) and intermediate (37.1%). Approximately 27.8% of participants had participated in specialty training, and 46.1% were involved in department management. Most head nurses were female (95.9%), and 39.9% had 16 years or more of head nurse experience. Monthly income was mainly concentrated between 4,000 and 8,000 CNY (51.2%), followed by 8,001–12,000 CNY (36.3%). Most participants reported neutral satisfaction with income (58.4%) and job satisfaction (62.0%), while 30.7% were dissatisfied with income and 30.6% were satisfied with their job.

**Table 1:** Demographic and work-related characteristics of participants (*n* = 765).

Variable	*n* (%)/mean ±SD
Gender	
Male	38 (5.0%)
Female	727 (95.0%)
Age (years)	33.45 ± 8.06
Marital status	
Unmarried	237 (31.0%)
Married	510 (66.7%)
Other status	18 (2.4%)
Birthplace	
Urban	275 (35.9%)
Rural	490 (64.1%)
Only child status	
Yes	348 (45.5%)
No	417 (54.5%)
Father's education level	
Primary school or below	126 (16.5%)
Junior high school	344 (45.0%)
Senior high school	207 (27.1%)
Associate degree or above	88 (11.5%)
**Mother's education level**	
Primary school or below	211 (27.6%)
Junior high school	336 (43.9%)
Senior high school	166 (21.7%)
Associate degree or above	52 (6.8%)
Parental overprotection/control	
Yes	165 (21.6%)
No	600 (78.4%)
Personality type	
Extrovert	124 (16.2%)
Introvert	181 (23.7%)
Ambivert	460 (60.1%)
Educational level	
Senior high school/secondary vocational school	4 (0.5%)
Associate degree	88 (11.5%)
Bachelor's degree	646 (84.4%)
Master's degree or above	27 (3.5%)
Years of working experience (years)	11.67 ± 8.81
Professional title	
Junior	382 (49.9%)
Intermediate	284 (37.1%)
Senior	99 (12.9%)
Participation in specialty training	
Yes	213 (27.8%)
No	552 (72.2%)
Involvement in department management	
Yes	353 (46.1%)
No	412 (53.9%)
Gender of head nurse	
Male	31 (4.1%)
Female	734 (95.9%)
Years of head nurse experience (years)	
≤ 5	158 (20.7%)
6–10	155 (20.3%)
11–15	147 (19.2%)
≥16	305 (39.9%)
Monthly income (CNY)	
< 4,000	66 (8.6%)
4,000–8,000	392 (51.2%)
8,001–12,000	278 (36.3%)
>12,000	29 (3.8%)
Income satisfaction	
Dissatisfied	235 (30.7%)
Neutral	447 (58.4%)
Satisfied	83 (10.8%)
Job satisfaction	
Dissatisfied	57 (7.5%)
Neutral	474 (62.0%)
Satisfied	234 (30.6%)

SD, standard deviation.

### 3.2 Latent profile analysis and naming of emotional intelligence among nurses

Latent profile models with one to five classes were estimated to determine the optimal solution. As shown in Table 2, the AIC, BIC, and aBIC values decreased as the number of classes increased, and the BLRT remained significant across the candidate models, indicating that solutions with additional classes remained statistically plausible. All candidate models converged normally, and the best log-likelihood values were replicated, reducing concern that the solutions reflected local maxima. The LMR test supported the 3-class model over the 2-class model (*P* = 0.036), whereas the 4-class model did not significantly improve model fit compared with the 3-class model (*P* = 0.524). The 5-class model was also not supported by the LMR test and produced a class below the 5% threshold. The 3-class model showed good classification accuracy, with an entropy of 0.917 and average posterior probabilities of 0.964, 0.960, and 0.974, respectively. In contrast, the 4-class and 5-class models mainly divided existing patterns into narrower subgroups and did not yield additional classes with clearer theoretical or clinical meaning. From a clinical nursing management perspective, the 3-class solution offered a parsimonious and interpretable framework that may help inform targeted intervention strategies. Therefore, the 3-class model was retained as the optimal solution.

**Table 2:** Model fit indices for latent profile analysis of emotional intelligence (1–5 classes).

Model	LL	AIC	BIC	aBIC	Entropy	LMR (*P*)	BLRT (*P*)	Proportion
1	−14,721.677	29,507.354	29,655.830	29,554.216	–	–	–	–
2	−13,209.761	26,517.521	26,744.875	26,589.279	0.902	< 0.001	< 0.001	0.532/0.468
3	**−12,800.436**	**25,732.873**	**26,039.105**	**25,829.525**	**0.917**	**0.036**	**< 0.001**	**0.417/0.508/0.075**
4	−12,448.484	25,062.969	25,448.079	25,184.517	0.910	0.524	< 0.001	0.195/0.412/0.303/0.090
5	−12,114.178	24,428.356	24,892.344	24,574.799	0.942	0.203	< 0.001	0.049/0.365/0.358/0.161/0.067

Bold values indicate the retained 3-class model selected as the optimal solution. aBIC, sample-size adjusted Bayesian Information Criterion; LMR, Lo-Mendell-Rubin adjusted likelihood ratio test.

AIC, Akaike information criterion; BIC, Bayesian information criterion; BLRT, bootstrap likelihood ratio test; LL, log-likelihood.

**Figure 1:**
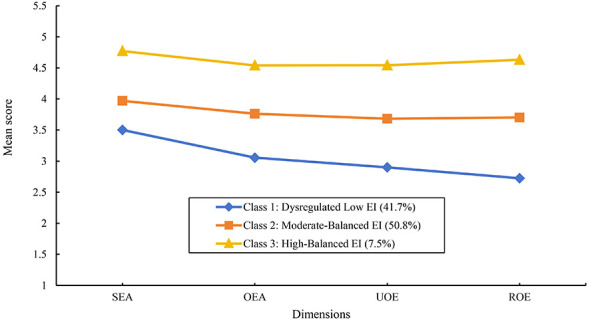
Latent profile analysis of emotional intelligence: Distribution across four dimensions in three profiles among clinical nurses. SEA, self-emotional appraisal; OEA, others' emotional appraisal; UOE, use of emotion; ROE, regulation of emotion. Class 1: Dysregulated Low emotional intelligence; Class 2: Moderate-Balanced emotional intelligence; Class 3: High-Balanced emotional intelligence.

The characteristics and labels of the three profiles were determined based on the distribution of scores across the four EI dimensions (Figure 1, Table 3). Class 1 accounted for 41.7% (*n* = 319) of the participants. This profile showed a downward pattern across dimensions: scores were relatively moderate for self-emotional appraisal (mean = 3.50) but lower for use of emotion (mean = 2.90) and regulation of emotion (mean = 2.72). This pattern was characterized by relatively higher self-emotional appraisal and lower scores for use and regulation of emotion. Therefore, this profile was labeled the “Dysregulated Low EI.” Class 2 accounted for 50.8% (*n* = 389) of the participants and displayed a relatively flat profile, with intermediate scores ranging from 3.68 to 3.97 across all dimensions. Because this profile showed moderate and relatively even scores across dimensions, it was labeled “Moderate-Balanced EI.” Class 3 accounted for 7.5% (*n* = 57) of the participants and was characterized by high scores across all dimensions (mean > 4.50). Reflecting this high and balanced score pattern, this profile was labeled “High-Balanced EI.”

**Table 3:** Comparison of emotional intelligence in the three-profile model (*n* = 765).

Variable	Class 1 (*n* = 319)	Class 2 (*n* = 389)	Class 3 (*n* = 57)	*F*	*P*-value	Bonferroni *post-hoc*
	Mean ±**SD**	**Mean** ±**SD**	**Mean** ±**SD**			
Total score	3.05 ± 0.32	3.78 ± 0.21	4.62 ± 0.23	1,184.743	< 0.001	1 < 2 < 3
Self-emotional appraisal (SEA)	3.50 ± 0.57	3.97 ± 0.37	4.77 ± 0.36	213.173	< 0.001	1 < 2 < 3
Others' emotional appraisal (OEA)	3.06 ± 0.61	3.76 ± 0.51	4.54 ± 0.57	244.341	< 0.001	1 < 2 < 3
Use of emotion (UOE)	2.90 ± 0.58	3.68 ± 0.45	4.54 ± 0.48	357.935	< 0.001	1 < 2 < 3
Regulation of emotion (ROE)	2.72 ± 0.53	3.70 ± 0.45	4.63 ± 0.47	562.418	< 0.001	1 < 2 < 3

Class 1, Dysregulated Low Emotional Intelligence; Class 2, Moderate-Balanced Emotional Intelligence; Class 3, High-Balanced Emotional Intelligence.

SD, standard deviation.

The comparison of EI scores among the three latent profiles is summarized in Table 3. The results of the one-way ANOVA indicated statistically significant differences in the total score and all four dimensions among the three profiles (*P* < 0.001). Bonferroni *post hoc* tests further showed a hierarchical pattern across the EI dimensions, with the High-Balanced EI profile scoring significantly higher than the Moderate-Balanced EI profile, followed by the Dysregulated Low EI profile (*P* < 0.001). Overall, these results indicated statistically significant and ordered differences across the three profiles in the total EI score and all four dimensions.

### 3.3 Univariate analysis of emotional intelligence profiles among nurses

Univariate analysis was conducted to compare the demographic and work-related characteristics among the three latent profiles (Table 4). Significant differences were observed in age (*P* = 0.035), parental overprotection or control (*P* < 0.001), and personality type (*P* < 0.001). Class 1 had the highest percentage of participants with a history of parental overprotection or control (28.8%) and those identifying as introverts (33.9%). Class 2 was predominantly composed of ambiverts (64.8%), while Class 3 contained the highest proportion of extroverts (26.3%). Regarding work-related characteristics, significant differences were found in years of working experience (*P* = 0.031), professional title (*P* = 0.008), involvement in department management (*P* < 0.001), income satisfaction (*P* = 0.010), and job satisfaction (*P* < 0.001). Class 1 reported the lowest rate of management involvement (29.5%) and the highest rates of dissatisfaction with both income (35.7%) and job (12.2%). Class 2 exhibited intermediate characteristics, with 55.8% involved in department management and 36.0% reporting satisfaction with their job. Class 3 was characterized by the highest rate of involvement in department management (73.7%) and the highest proportion of job satisfaction (54.4%).

**Table 4:** Univariate analysis of emotional intelligence profiles (*n* = 765).

Variable	Class 1 (*n* = 319)	Class 2 (*n* = 389)	Class 3 (*n* = 57)	Test statistic	*P*-value
	***n*** **(%)/mean** ±**SD**	***n*** **(%)/mean** ±**SD**	***n*** **(%)/mean** ±**SD**		
Gender					
Male	13 (4.1%)	22 (5.7%)	3 (5.3%)	0.939^a^	0.625
Female	306 (95.9%)	367 (94.3%)	54 (94.7%)		
Age (years)	32.76 ± 7.96	33.78 ± 8.17	34.95 ± 7.60	6.678^b^	0.035^*^
Marital status					
Unmarried	109 (34.2%)	111 (28.5%)	17 (29.8%)	5.436^a^	0.245
Married	200 (62.7%)	270 (69.4%)	40 (70.2%)		
Other status	10 (3.1%)	8 (2.1%)	0 (0.0%)		
Birthplace					
Urban	104 (32.6%)	153 (36.3%)	18 (31.6%)	3.958^a^	0.138
Rural	215 (67.4%)	236 (60.7%)	39 (68.4%)		
Only child status					
Yes	138 (43.3%)	189 (48.6%)	21 (36.8%)	3.863^a^	0.145
No	181 (56.7%)	200 (51.4%)	36 (63.2%)		
Father's education level					
Primary school or below	53 (16.6%)	65 (16.7%)	8 (14.0%)	3.262^b^	0.196
Junior high school	156 (48.9%)	166 (42.7%)	22 (38.6%)		
Senior high school	78 (24.5%)	110 (28.3%)	19 (33.3%)		
Associate degree or above	32 (10.0%)	48 (12.3%)	8 (14.0%)		
**Mother's education level**					
Primary school or below	89 (27.9%)	108 (27.8%)	14 (24.6%)	0.078^b^	0.962
Junior high school	139 (43.6%)	167 (42.9%)	30 (52.6%)		
Senior high school	70 (21.9%)	87 (22.4%)	9 (15.8%)		
Associate degree or above	21 (6.6%)	27 (6.9%)	4 (7.0%)		
Parental overprotection/control					
Yes	92 (28.8%)	66 (17.0%)	7 (12.3%)	17.748^a^	< 0.001^**^
No	227 (71.2%)	323 (83.0%)	50 (87.7%)		
Personality type					
Extrovert	35 (11.0%)	74 (19.0%)	15 (26.3%)	37.823^a^	< 0.001^**^
Introvert	108 (33.9%)	63 (16.2%)	10 (17.5%)		
Ambivert	176 (55.2%)	252 (64.8%)	32 (56.1%)		
Educational level					
Senior high school/secondary vocational school	2 (0.6%)	2 (0.5%)	0 (0.0%)	2.432^b^	0.296
Associate degree	44 (13.8%)	38 (9.8%)	6 (10.5%)		
Bachelor's degree	262 (82.1%)	336 (86.4%)	48 (84.2%)		
Master's degree or above	11 (3.4%)	13 (3.3%)	3 (5.3%)		
Years of working experience (years)	10.87 ± 8.76	12.15 ± 8.94	12.81 ± 8.03	6.957^b^	0.031^*^
Professional title					
Junior	173 (54.2%)	186 (47.8%)	23 (40.4%)	9.741^b^	0.008^*^
Intermediate	121 (37.9%)	142 (36.5%)	21 (36.8%)		
Senior	25 (7.8%)	61 (15.7%)	13 (22.8%)		
Participation in specialty training					
Yes	75 (23.5%)	123 (31.6%)	15 (26.3%)	5.807^a^	0.055
No	244 (76.5%)	266 (68.4%)	42 (73.7%)		
Involvement in department management					
Yes	94 (29.5%)	217 (55.8%)	42 (73.7%)	67.643^a^	< 0.001^**^
No	225 (70.5%)	172 (44.2%)	15 (26.3%)		
Gender of head nurse					
Male	12 (3.8%)	17 (4.4%)	2 (3.5%)	0.214^a^	0.899
Female	307 (96.2%)	372 (95.6%)	55 (96.5%)		
Years of head nurse experience (years)					
≤ 5	64 (20.1%)	84 (21.6%)	10 (17.5%)	0.107^b^	0.948
6–10	67 (21.0%)	75 (19.3%)	13 (22.8%)		
11–15	60 (18.8%)	76 (19.5%)	11 (19.3%)		
≥16	128 (40.1%)	154 (39.6%)	23 (40.4%)		
Monthly income (CNY)					
< 4,000	32 (10.0%)	32 (8.2%)	2 (3.5%)	4.672^b^	0.097
4,000–8,000	165 (51.7%)	201 (51.7%)	26 (45.6%)		
8,001–12,000	114 (35.7%)	138 (35.5%)	26 (45.6%)		
>12,000	8 (2.5%)	18 (4.2%)	3 (5.3%)		
Income satisfaction					
Dissatisfied	114 (35.7%)	102 (26.2%)	19 (33.3%)	9.161^b^	0.010^*^
Neutral	180 (56.4%)	241 (62.0%)	26 (45.6%)		
Satisfied	25 (7.8%)	46 (11.8%)	12 (21.1%)		
Job satisfaction					
Dissatisfied	39 (12.2%)	13 (3.3%)	5 (8.8%)	43.399^b^	< 0.001^**^
Neutral	217 (68.0%)	236 (60.7%)	21 (36.8%)		
Satisfied	63 (19.7%)	140 (36.0%)	31 (54.4%)		

Class 1, Dysregulated Low Emotional Intelligence; Class 2, Moderate-Balanced Emotional Intelligence; Class 3, High-Balanced Emotional Intelligence.

^a^Chi-square value.

^b^H-value.

^**^P < 0.001.

^*^P < 0.05.

SD, standard deviation.

### 3.4 Multinomial logistic regression analysis of emotional intelligence profiles among nurses

Multinomial logistic regression analysis was used to identify factors associated with latent profile membership, with Class 1 serving as the reference category. As shown in Table 5, parental overprotection or control, personality type, involvement in department management, and job satisfaction were significantly associated with profile membership. Nurses who reported parental overprotection or control had lower odds of belonging to Class 2 rather than Class 1 (OR = 0.621, 95% CI: 0.421–0.915). Compared with ambivert nurses, introverted nurses also had lower odds of belonging to Class 2 (OR = 0.441, 95% CI: 0.299–0.652). Nurses involved in department management had higher odds of belonging to Class 2 (OR = 2.624, 95% CI: 1.815–3.794) and Class 3 (OR = 6.112, 95% CI: 2.932–12.744). Compared with nurses who were satisfied with their job, those who were dissatisfied (OR = 0.180, 95% CI: 0.083–0.392) or neutral (OR = 0.510, 95% CI: 0.341–0.762) had lower odds of belonging to Class 2. Neutral job satisfaction was also associated with lower odds of belonging to Class 3 (OR = 0.215, 95% CI: 0.106–0.438). Age, professional title, and income satisfaction were not significantly associated with profile membership.

**Table 5:** Multinomial logistic regression of emotional intelligence profiles (*n* = 765).

Variable	Class 2: Moderate-Balanced EI (ref. class 1: Dysregulated Low EI)						Class 3: High-Balanced EI (ref. class 1: Dysregulated Low EI)					
	**B**	**SE**	**Wald** χ^2^	***P*** **-value**	**OR**	**95% CI**	**B**	**SE**	**Wald** χ^2^	***P*** **-value**	**OR**	**95% CI**
Age (years)	−0.026	0.015	2.829	0.093	0.974	0.945–1.004	−0.042	0.031	1.907	0.167	0.958	0.902–1.018
Parental overprotection/control (ref. no)												
Yes	−0.477	0.198	5.813	0.016^*^	0.621	0.421–0.915	−0.770	0.443	3.027	0.082	0.463	0.194–1.102
Personality type (ref. ambivert)												
Extrovert	0.268	0.241	1.240	0.265	1.308	0.816–2.097	0.653	0.388	2.836	0.092	1.921	0.899–4.105
Introvert	−0.818	0.199	16.860	< 0.001^**^	0.441	0.299–0.652	−0.501	0.407	1.517	0.218	0.606	0.273–1.345
Professional title (ref. senior)												
Junior	−0.422	0.377	1.256	0.262	0.656	0.314–1.372	−0.789	0.654	1.455	0.228	0.454	0.126–1.637
Intermediate	−0.310	0.306	1.029	0.310	0.733	0.403–1.335	−0.574	0.488	1.379	0.240	0.563	0.216–1.468
Involvement in department management (ref. no)												
Yes	0.965	0.188	26.289	< 0.001^**^	2.624	1.815–3.794	1.810	0.375	23.319	< 0.001^**^	6.112	2.932–12.744
Income satisfaction (ref. satisfied)												
Dissatisfied	−0.005	0.340	0.000	0.988	0.995	0.511–1.935	0.163	0.539	0.091	0.763	1.177	0.409–3.383
Neutral	0.000	0.306	0.000	1.000	1.000	0.549–1.821	−0.522	0.465	1.257	0.262	0.594	0.238–1.477
Job satisfaction (ref. satisfied)												
Dissatisfied	−1.716	0.398	18.630	< 0.001^**^	0.180	0.083–0.392	−1.215	0.626	3.774	0.052	0.297	0.087–1.011
Neutral	−0.674	0.205	10.758	0.001^*^	0.510	0.341–0.762	−1.536	0.363	17.935	< 0.001^**^	0.215	0.106–0.438

^**^P < 0.001.

^*^P < 0.05.

CI, confidence interval; EI, emotional intelligence; OR, odds ratio; SE, standard error.

Because of severe multicollinearity between age and years of working experience, age was retained in the primary model, and years of working experience was examined in a sensitivity analysis. The results were largely consistent with the primary model (Supplementary Table S2). Years of working experience was not significantly associated with membership in Class 2 (OR = 0.977, 95% CI: 0.951–1.004). However, it showed a significant negative association with membership in Class 3 (OR = 0.941, 95% CI: 0.889–0.996), indicating that longer working experience was associated with lower odds of belonging to Class 3 rather than Class 1. These findings suggest that the main regression results were robust to the choice between these two collinear variables.

A sensitivity analysis evaluated the robustness of the primary findings to classification uncertainty. Excluding participants with a maximum posterior probability below 0.80 yielded a high-certainty subsample of 712 individuals, retaining 93.1% of the original cohort. The profile distribution within this refined group remained consistent with the primary analysis: Class 1 (*n* = 297, 41.7%), Class 2 (*n* = 360, 50.6%), and Class 3 (*n* = 55, 7.7%). The multinomial logistic regression model was then re-estimated in this subsample (Supplementary Table S3). The overall pattern of associations was largely consistent with the primary model. Involvement in department management, job satisfaction, and introverted personality retained their significant associations with latent profile membership, with unchanged directions and comparable estimates. Age, professional title, and income satisfaction remained non-significant. In the high-certainty subsample, parental overprotection or control and job dissatisfaction reached statistical significance for Class 3 (OR = 0.371, 95% CI: 0.147–0.933; OR = 0.286, 95% CI: 0.083–0.985, respectively). These results suggest that the main findings were not substantially influenced by classification uncertainty.

## 4 Discussion

This study identified three empirical EI profiles among clinical nurses: Dysregulated Low EI, Moderate-Balanced EI, and High-Balanced EI. The largest proportion of nurses showed a moderate and relatively balanced level of EI, which is consistent with previous findings among nurses (32). The use of LPA helped reveal meaningful heterogeneity in EI patterns that may be obscured when only total scores are used. At the same time, the differences among profiles appeared to be largely gradual across the EI dimensions, and the profile labels should therefore be understood as descriptions of dominant response patterns. Consistent with previous findings (33), nurses across all three profiles showed lower scores in others' emotional appraisal, use of emotion, and regulation of emotion than in self-emotional appraisal. From a theoretical perspective, effective emotion regulation and emotion use require more complex cognitive processing than basic emotional awareness (34, 35). Under sustained emotional labor, depleted psychological resources may make it more difficult for nurses to regulate and use emotions effectively, even when they are aware of their own emotional states (36). Multinomial logistic regression identified parental overprotection or control, personality type, involvement in department management, and job satisfaction as factors associated with EI profile membership, providing a person-centered basis for targeted intervention strategies.

The Dysregulated Low EI profile showed an imbalanced pattern, with relatively higher self-emotional appraisal but lower scores in others' emotional appraisal, use of emotion, and regulation of emotion. This pattern suggests that nurses in this profile may be relatively more aware of their own emotions while having greater difficulty using and regulating emotions in demanding clinical situations. Such an imbalance may be associated with greater vulnerability to burnout and challenges in maintaining humanistic care, particularly under sustained emotional labor (10, 37). These patterns may be associated with poorer coping under heavy workloads and emotional labor (38), with potential implications for nurses' wellbeing and the quality of nursing care (13, 39).

The Moderate-Balanced EI profile showed relatively even but moderate scores across all dimensions, suggesting potential room for further development (40). For nurses in this profile, situational factors such as job demands, organizational support, and work environment may be associated with their EI levels (41, 42). Reducing excessive workloads, strengthening support, and creating a positive work environment may help maintain or improve EI among nurses with moderate and relatively balanced scores.

The High-Balanced EI profile showed high and relatively even scores across all dimensions, suggesting a more favorable pattern of perceived emotional functioning. However, this profile accounted for only 7.5% of the total sample. This finding suggests that nurses in this profile may serve as a potential source of peer support within the nursing workforce. Nursing managers may consider involving them as peer supporters to provide emotional guidance and support for other nurses (43, 44). Specifically, they can pair nurses with high EI with those who have relatively lower EI to provide emotional guidance and support. Such peer support strategies may help create a supportive environment for the development of EI (45).

Nurses who experienced parental overprotection or control during their early years were more likely to belong to the Dysregulated Low EI profile. Parental overprotection has been associated with less adaptive emotional processing and emotion regulation strategies (46, 47). These early experiences may be related to heightened sensitivity to personal distress and less adaptive emotion regulation under clinical pressure (48). Such difficulties may be associated with challenges in responding to patients' emotional needs and maintaining humanistic care (49). For nurses who experience these difficulties, supportive interventions may focus on adaptive emotion processing and emotion regulation. Mindfulness training has been shown to improve emotion regulation by cultivating nonreactive awareness of emotions and reducing experiential avoidance (50, 51). Nursing managers may incorporate mindfulness training into routine programs to support emotion regulation and more adaptive emotion use.

Nurses who self-identified as introverted were more likely to belong to the Dysregulated Low EI profile. One possible interpretation is that these nurses may be more inclined to internalize occupational stress or use less outward emotional expression, which may be reflected in greater expressive suppression (52). Expressive suppression has been described as a less adaptive emotion regulation strategy that may compromise effective emotional regulation and is relevant to lower EI (52). Salem et al. (53) showed that reflective mindfulness and emotional regulation training significantly improved cognitive reappraisal and reduced expressive suppression among nursing students, suggesting its potential to enhance emotional expression and regulation. Nursing managers may integrate these approaches into routine training programs to support nurses' emotional expression and regulation.

Nurses who participated in department management activities were more likely to belong to the High-Balanced EI profile. Participation in management-related activities requires continuous interpersonal coordination, communication, and conflict resolution, which are closely aligned with key dimensions of EI, such as others' emotional appraisal and emotion regulation (54). Previous research indicates that participatory management practices are associated with enhanced nurses' professional competence (55). Therefore, involving frontline nurses in management practices such as mentoring and quality control may create practical opportunities for them to use and further develop EI while supporting professional growth.

Nurses with low job satisfaction were more likely to belong to the Dysregulated Low EI profile. Low job satisfaction may reflect heavy workloads, limited support, and an unfavorable work environment, which may deplete psychological resources and be associated with lower EI scores (41, 56). This finding aligns with the Job Demands-Resources model (57). Previous research shows that enhancing organizational resources helps buffer the negative impact of job demands on personal resources, while also supporting nurses in maintaining stable emotional functioning in clinical settings (18, 44). Therefore, nursing managers may optimize human resource allocation and strengthen organizational support to protect nurses' psychological resources and support the development of EI.

In the sensitivity analysis, nurses with longer working experience were less likely to be in the High-Balanced EI profile than in the Dysregulated Low EI profile. This finding suggests that the relationship between clinical experience and EI may not follow a simple linear pattern. Clinical experience may provide opportunities for nurses to accumulate practical knowledge, strengthen communication competence, and develop more adaptive responses to emotional demands (58, 59). However, prolonged exposure to heavy workloads and emotional labor may also strain emotional resources in tertiary hospital settings. Previous studies have reported inconsistent associations between nursing experience and EI across different career stages (60, 61). These findings suggest that EI development should be supported across different career stages, as years of practice alone may not ensure the development of EI.

### 4.1 Clinical implications for nursing management

The findings suggest that EI should be incorporated into routine nursing workforce development in comparable tertiary hospital settings. The identified profiles may help nursing managers recognize heterogeneity in nurses' EI and plan differentiated support as part of routine departmental management. Such support can include periodic assessment of EI patterns, training grounded in clinical scenarios for emotional appraisal, emotion use, and emotion regulation, and ongoing guidance for nurses facing sustained emotional labor. To sustain EI development, nursing managers may foster a supportive practice environment through structured team coordination and peer reflection for nurses with high emotional labor demands. These strategies may contribute to nurses' wellbeing and the quality of nursing care.

### 4.2 Limitations

This study has several limitations. First, the cross-sectional design limits causal inference and prevents determination of the directionality of associations between related factors and EI profiles. Future longitudinal studies are needed to examine the stability and developmental trajectories of these profiles. Second, although confirmatory factor analysis supported the four-factor structure of the WLEIS in this sample, EI was assessed using a self-reported scale and may reflect perceived EI rather than objective emotional ability. Self-reported EI may also be influenced by self-perception, social desirability, and professional identity among nurses. Some associated factors, including parental overprotection/control, personality type, income satisfaction, and job satisfaction, were measured using single self-developed items, which may introduce response bias and measurement error. Future studies should consider combining ability-based EI measures with self-report instruments and using widely adopted, psychometrically validated measures for these associated factors to enhance the reliability and validity of the findings. Third, all variables were collected through participant questionnaires at a single time point, which may increase the risk of common method bias. Although the ULMC assessment showed only small changes in model fit, it cannot fully rule out this possibility. Future studies should incorporate data from multiple sources and behavioral indicators to reduce common method bias. Fourth, although the three-profile model showed high classification accuracy and the sensitivity analysis supported the robustness of the regression results, the regression analysis was still based on the most likely class assignment. Future studies may consider three-step approaches, such as R3STEP, to further account for classification uncertainty when examining factors associated with latent profile membership. Fifth, the sample was recruited from two tertiary Grade A hospitals within a single Chinese city, and most participants were female. Although this gender distribution reflects the current structure of the nursing workforce, the findings may not be directly generalizable to nurses in other regions, lower-level healthcare institutions, different healthcare systems, or cultural contexts. Future multicenter studies across diverse geographic regions and healthcare settings are needed to further examine the generalizability of the identified EI profiles and their associated factors.

## 5 Conclusions

This study identified three EI profiles among clinical nurses in tertiary Grade A hospitals: Dysregulated Low EI (41.7%), Moderate-Balanced EI (50.8%), and High-Balanced EI (7.5%). The majority of nurses (50.8%) showed moderate and relatively balanced EI scores across dimensions. We found that parental overprotection or control, personality type, involvement in department management, and job satisfaction were associated with profile membership. These findings highlight the heterogeneity of EI within the nursing workforce. The identified profiles may inform differentiated management strategies and targeted training programs for nurses with different EI levels. Such tailored strategies may help support nurses' mental wellbeing and contribute to the quality of nursing care.

## Data Availability

The original contributions presented in the study are included in the article/Supplementary material, further inquiries can be directed to the corresponding author.
