# Supplementary material for: Latent profiles of emotional intelligence and associated factors among clinical nurses: a cross-sectional study
Source: Front Public Health. 2026 Jun 12;14:1851059. doi: 10.3389/fpubh.2026.1851059 (PMC13303749; doi:10.3389/fpubh.2026.1851059)
Supplement: Supplementary file 1 [file table_1.docx]

**Supplementary Table S1. Measurement and coding of key self-developed predictor variables.**

| **Variable** | **Original item wording** | **Item wording** | **Response options** | **Coding and reference category** | **Rationale** |
| --- | --- | --- | --- | --- | --- |
| Parental overprotection/control | 您觉得您的人生当中父母是否存在强烈控制或过度保护？ | In your view, did your parents show strong control or overprotection toward you during your upbringing? | Yes; No | Binary variable: No = 0, Yes = 1; reference category in multinomial logistic regression: No. | This item was included to capture nurses’ self-reported experience of parental overprotection or control, as early family environment may be related to emotional processing and emotion regulation in adulthood. |
| Personality type | 您的性格类型为？ | Which of the following best describes your personality type? | Extrovert; Introvert; Ambivert | Nominal categorical variable: Extrovert = 1, Introvert = 2, Ambivert = 3; reference category in multinomial logistic regression: Ambivert. | This item was included to describe nurses’ self-identified personality tendency, which may be related to emotional expression, interpersonal communication, and EI profiles. |
| Involvement in department management | 是否协助（或曾协助）科室管理（如担任护理组长、带教组长、院感组长等）？ | Are you currently involved, or have you previously been involved, in department management activities, such as serving as a nursing team leader, teaching group leader, or infection control group leader? | Yes; No | Binary variable: No = 0, Yes = 1; reference category in multinomial logistic regression: No. | This item was included to reflect nurses’ participation in department-level management activities, which may be associated with communication, interpersonal coordination, and EI-related functioning. |
| Income satisfaction | 您对个人月收入的满意度？ | How satisfied are you with your personal monthly income? | Dissatisfied; Neutral;  Satisfied | Ordinal categorical variable: Dissatisfied = 1, Neutral = 2, Satisfied = 3; reference category in multinomial logistic regression: Satisfied. | This item was included to reflect nurses’ subjective evaluation of income, which may be related to work attitudes and psychological resources. |
| Job satisfaction | 您对护理工作的满意度？ | How satisfied are you with your nursing work? | Dissatisfied; Neutral;  Satisfied | Ordinal categorical variable: Dissatisfied = 1, Neutral = 2, Satisfied = 3; reference category in multinomial logistic regression: Satisfied. | This item was included to capture nurses’ overall satisfaction with their nursing work, a work-related psychological factor that may be associated with emotional functioning and EI profiles. |

Note*.* Numeric codes were used for data entry. All variables listed in this table were entered as categorical predictors in the multinomial logistic regression, with the reference categories shown above.
Abbreviations: EI, Emotional Intelligence.
