# Supplementary material for: Latent profiles of emotional intelligence and associated factors among clinical nurses: a cross-sectional study
Source: Front Public Health. 2026 Jun 12;14:1851059. doi: 10.3389/fpubh.2026.1851059 (PMC13303749; doi:10.3389/fpubh.2026.1851059)
Supplement: Supplementary file 2 [file table_2.docx]

**Supplementary Table S2. Multinomial logistic regression of emotional intelligence profiles: Sensitivity analysis replacing age with years of working experience (*n* = 765).**

| **Variable** | **Class 2: Moderate-Balanced EI (ref. Class 1: Dysregulated Low EI)** | | | | | | **Class 3: High-Balanced EI (ref. Class 1: Dysregulated Low EI)** | | | | | |
| --- | --- | --- | --- | --- | --- | --- | --- | --- | --- | --- | --- | --- |
|  | **B** | **SE** | **Wald** $\boldsymbol{\chi}^{\boldsymbol{2}}$ | ***P*** | **OR** | **95% CI** | **B** | **SE** | **Wald** $\boldsymbol{\chi}^{\boldsymbol{2}}$ | ***P*** | **OR** | **95% CI** |
| **Parental overprotection/control (ref. No)** |  |  |  |  |  |  |  |  |  |  |  |  |
| Yes | $-$0.474 | 0.198 | 5.749 | 0.016* | 0.622 | 0.422–0.917 | $-$0.767 | 0.443 | 2.995 | 0.083 | 0.464 | 0.195–1.107 |
| **Personality type (ref. Ambivert)** |  |  |  |  |  |  |  |  |  |  |  |  |
| Extrovert | 0.278 | 0.241 | 1.334 | 0.248 | 1.321 | 0.824–2.119 | 0.677 | 0.388 | 3.038 | 0.081 | 1.968 | 0.919–4.213 |
| Introvert | $-$0.825 | 0.199 | 17.141 | < 0.001** | 0.438 | 0.297–0.648 | $-$0.520 | 0.408 | 1.625 | 0.202 | 0.594 | 0.267–1.323 |
| **Years of working experience (years)** | $-$0.023 | 0.014 | 2.720 | 0.099 | 0.977 | 0.951–1.004 | $-$0.061 | 0.029 | 4.383 | 0.036* | 0.941 | 0.889–0.996 |
| **Professional title (ref. Senior)** |  |  |  |  |  |  |  |  |  |  |  |  |
| Junior | $-$0.414 | 0.377 | 1.205 | 0.272 | 0.661 | 0.316–1.384 | $-$1.143 | 0.662 | 2.985 | 0.084 | 0.319 | 0.087–1.166 |
| Intermediate | $-$0.317 | 0.307 | 1.063 | 0.303 | 0.728 | 0.399–1.330 | $-$0.742 | 0.498 | 2.220 | 0.136 | 0.476 | 0.179–1.264 |
| **Involvement in department management (ref. No)** |  |  |  |  |  |  |  |  |  |  |  |  |
| Yes | 0.969 | 0.189 | 26.374 | < 0.001** | 2.635 | 1.821–3.815 | 1.856 | 0.376 | 24.375 | < 0.001** | 6.396 | 3.062–13.360 |
| **Income satisfaction (ref. Satisfied)** |  |  |  |  |  |  |  |  |  |  |  |  |
| Dissatisfied | $-$0.013 | 0.341 | 0.002 | 0.968 | 0.987 | 0.506–1.925 | 0.036 | 0.545 | 0.004 | 0.947 | 1.037 | 0.357–3.015 |
| Neutral | $-$0.009 | 0.307 | 0.001 | 0.976 | 0.991 | 0.543–1.808 | $-$0.616 | 0.470 | 1.717 | 0.190 | 0.540 | 0.215–1.357 |
| **Job satisfaction (ref. Satisfied)** |  |  |  |  |  |  |  |  |  |  |  |  |
| Dissatisfied | $-$1.722 | 0.397 | 18.771 | < 0.001** | 0.179 | 0.082–0.390 | $-$1.202 | 0.627 | 3.667 | 0.056 | 0.301 | 0.088–1.029 |
| Neutral | $-$0.674 | 0.206 | 10.736 | 0.001* | 0.510 | 0.341–0.763 | $-$1.528 | 0.364 | 17.661 | < 0.001** | 0.217 | 0.106–0.442 |

Note. ^**^ *P* < 0.001 ^*^ *P* < 0.05.

Abbreviations: CI, Confidence Interval; EI, Emotional Intelligence; OR, Odds Ratio; SE, Standard Error.
