# Supplementary material for: Latent profiles of emotional intelligence and associated factors among clinical nurses: a cross-sectional study
Source: Front Public Health. 2026 Jun 12;14:1851059. doi: 10.3389/fpubh.2026.1851059 (PMC13303749; doi:10.3389/fpubh.2026.1851059)
Supplement: Supplementary file 3 [file table_3.docx]

**Supplementary Table S3. Multinomial logistic regression of emotional intelligence profiles: Sensitivity analysis within the high-certainty subsample (*n* = 712).**

| **Variable** | **Class 2: Moderate-Balanced EI (ref. Class 1: Dysregulated Low EI)** | | | | | | **Class 3: High-Balanced EI (ref. Class 1: Dysregulated Low EI)** | | | | | |
| --- | --- | --- | --- | --- | --- | --- | --- | --- | --- | --- | --- | --- |
|  | **B** | **SE** | **Wald** $\boldsymbol{\chi}^{\boldsymbol{2}}$ | ***P*** | **OR** | **95% CI** | **B** | **SE** | **Wald** $\boldsymbol{\chi}^{\boldsymbol{2}}$ | ***P*** | **OR** | **95% CI** |
| **Age (years)** | $-$0.024 | 0.016 | 2.334 | 0.127 | 0.976 | 0.946–1.007 | $-$0.041 | 0.031 | 1.717 | 0.190 | 0.960 | 0.902–1.021 |
| **Parental overprotection/control (ref. No)** |  |  |  |  |  |  |  |  |  |  |  |  |
| Yes | $-$0.488 | 0.205 | 5.656 | 0.017* | 0.614 | 0.411–0.918 | $-$0.992 | 0.471 | 4.438 | 0.035* | 0.371 | 0.147–0.933 |
| **Personality type (ref. Ambivert)** |  |  |  |  |  |  |  |  |  |  |  |  |
| Extrovert | 0.387 | 0.252 | 2.361 | 0.124 | 1.473 | 0.899–2.413 | 0.713 | 0.403 | 3.134 | 0.077 | 2.040 | 0.927–4.492 |
| Introvert | $-$0.852 | 0.208 | 16.773 | < 0.001** | 0.427 | 0.284–0.641 | $-$0.441 | 0.410 | 1.156 | 0.282 | 0.643 | 0.288–1.438 |
| **Professional title (ref. Senior)** |  |  |  |  |  |  |  |  |  |  |  |  |
| Junior | $-$0.393 | 0.392 | 1.006 | 0.316 | 0.675 | 0.313–1.455 | $-$0.747 | 0.671 | 1.240 | 0.266 | 0.474 | 0.127–1.765 |
| Intermediate | $-$0.270 | 0.320 | 0.714 | 0.398 | 0.763 | 0.408–1.429 | $-$0.615 | 0.503 | 1.493 | 0.222 | 0.541 | 0.202–1.450 |
| **Involvement in department management (ref. No)** |  |  |  |  |  |  |  |  |  |  |  |  |
| Yes | 0.959 | 0.197 | 23.580 | < 0.001** | 2.609 | 1.772–3.842 | 1.820 | 0.384 | 22.493 | < 0.001** | 6.173 | 2.910–13.098 |
| **Income satisfaction (ref. Satisfied)** |  |  |  |  |  |  |  |  |  |  |  |  |
| Dissatisfied | 0.101 | 0.350 | 0.083 | 0.773 | 1.106 | 0.557–2.194 | 0.260 | 0.546 | 0.227 | 0.633 | 1.297 | 0.445–3.782 |
| Neutral | 0.039 | 0.314 | 0.016 | 0.900 | 1.040 | 0.562–1.924 | $-$0.572 | 0.476 | 1.445 | 0.229 | 0.564 | 0.222–1.435 |
| **Job satisfaction (ref. Satisfied)** |  |  |  |  |  |  |  |  |  |  |  |  |
| Dissatisfied | $-$1.816 | 0.412 | 19.423 | < 0.001** | 0.163 | 0.073–0.365 | $-$1.253 | 0.631 | 3.937 | 0.047* | 0.286 | 0.083–0.985 |
| Neutral | $-$0.677 | 0.213 | 10.066 | 0.002* | 0.508 | 0.335–0.772 | $-$1.503 | 0.372 | 16.297 | < 0.001** | 0.222 | 0.107–0.461 |

Note*.* ^**^ *P* < 0.001 ^*^ *P* < 0.05.

Abbreviations: CI, Confidence Interval; EI, Emotional Intelligence; OR, Odds Ratio; SE, Standard Error.
